# Molecular Spectroscopic (FTIR and UV-Vis) and Hyphenated Chromatographic (UHPLC-qTOF-MS) Analysis and *In Vitro* Bioactivities of the *Momordica balsamina* Leaf Extract

**DOI:** 10.1155/2021/2854217

**Published:** 2021-09-28

**Authors:** X. E. Mabasa, L. M. Mathomu, N. E. Madala, E. M. Musie, M. T. Sigidi

**Affiliations:** Department of Biochemistry and Microbiology, Faculty of Sciences,Agriculture and Engineering, University of Venda, Private Bag X5050, Thohoyandou, Limpopo, South Africa

## Abstract

*Momordica balsamina* (*M. balsamina*) is a medicinal herb comprising health-promoting secondary metabolites. This study was aimed to profile bioactive compounds in the methanolic extract of *M. balsamina* leaves using molecular spectroscopic (UV-Vis and FTIR) and hyphenated chromatographic (UHPLC-qTOF-MS) techniques and evaluate the biological (*in vitro* anti-inflammatory and cytotoxicity) activities of the extract. The preliminary phytochemical screening tests revealed the presence of cardiac glycosides, flavonoids, saponins, tannins, and terpenoids. The UV-Vis profile revealed various absorption bands ranging from 200 to 750 nm, indicating the presence of flavonoids, phenolic compounds, tannins, terpenoids, carotenoids, chlorophyll, and alkaloids. FTIR spectra confirmed the presence of alkaloids, flavonoids, terpenes, anthraquinones, and phenolic compounds. A high-resolution and accurate mass spectrometer (LC-QTOF-MS model LC-MS-9030 instrument) was used, and the results confirmed the presence of flavonoid aglycones, such as quercetin, isorhamnetin, and kaempferol, as well as pseudolaroside A and dicaffeoylquinic and feruloyl isocitric acids. To the best of our knowledge, this is the first report of pseudolaroside A dimer and feruloyl isocitric acid in *M. balsamina* leaves. *In vitro* cytotoxicity assay showed that the extract was nontoxic against human colorectal adenocarcinoma (HT29 and Caco2), Vero, and RAW 264.7 cells. However, the extract showed anti-inflammatory activity on RAW 264.7 cells. The study confirmed that *M. balsamina* leaves contain nontoxic secondary metabolites that may play a pivotal role in human health as anti-inflammatory agents.

## 1. Introduction

*Momordica balsamina (Cucurbitaceae)* is generally known as African cucumber or pumpkin, Balsam apple, or pear [1]. It is characterized by a bitter taste attributed to phytocompounds, such as alkaloids and cucurbitacins [2–4]. Other phytochemical studies have reported that this plant may also contain flavonoids, phenols, sterols, and anthraquinones [5, 6]. These have been reported to exhibit a wide range of biological activities such as anti-inflammatory [4], antiplasmodial [7], antidiabetic [8, 9], antidiarrheal [5, 10], antiviral [1, 5, 10], antibacterial [5, 10–14], and cardiovascular activity [2].

In certain regions of South Africa, these leaves are used as a remedy for sugar diabetes and chronic hypertension; however, this has no scientific backing [2]. In most villages, *Momordica* paste is used to eliminate intestinal worms in children and prevent irritation in anus. This is done by applying the paste externally on the anus as highlighted by Nagarani et al. [4]. *Momordica balsamina* has also shown the potential to treat gastroenteritis [13], strongly suggesting that these leaves may be used to treat gut-related infections. Therefore, plant profiling is crucial in order to link the phytocompounds with the biological activities of the plants.

Recently, the use of sophisticated techniques and scientific methods to profile and validate phytochemical compounds in medicinal plants has become more reliable [15]. In order for the herbal infusions to sustain quality, their safety status and effectiveness must meet the quality health standard [16]. A range of analytical methods can be used in assessing the degree of chemical purity of phytomedicines [16].

Chromatography and spectroscopy have become more effective and reliable tools used for phytochemical analysis [17]. Fourier transform infrared (FTIR) spectroscopy is used to characterize and identify functional groups [18]. Ultraviolet-visible spectrophotometry (UV-Vis) is related to photon spectroscopy in the UV-visible region [18, 19]. This technique uses light that is in the visible ranges of the electromagnetic spectrum [18, 19]. The colour of chemicals involved affects the absorption, and molecules undergo electron transition in these ranges [18].

Analysis of complex media using ultraviolet-visible (UV-Vis) spectroscopy is a disadvantage due to limitation by inherent difficulties when it comes to assigning peaks to any constituents in the system [20]. Therefore, the UV-Vis findings must be supplemented with other analytical techniques, such as GC-MS or LC-MS, for appropriate phytocompound profiling and constituent identification. Hence, in the present study, the extract was further subjected to hyphenated chromatographic technique (UHPLC-qTOF-MS) to identify the phytochemical constituents present in *M. balsamina*. The use of mass spectrometry (MS) in the development of plant metabolomics has made profiling multiple compounds, such as flavonoids and many more, possible [3, 21, 22].

The dependence on herbal concoctions prepared from plants, such as *M. balsamina*, has recently increased due to the severity and escalating burden of various diseases in humans. The continual usage and overharvesting of these herbs for medicinal purposes bring an urge to scientifically validate the biological effects these extracts might have [23].

Inflammation is a crucial and complex host's defensive mechanism that is intended to eliminate the initial cause of cell injury induced by microbial infections [24]. Initially, immune cells migrate from blood vessels and mediators, such as adhesion molecules, cytokines, and chemokines released at the site of damage [25]. Inflammatory cells are then recruited and reactive oxygen species (ROS), reactive nitrogen species (RNS), and proinflammatory cytokines are released to eradicate foreign pathogens and thus repair injured tissues [24, 25]. The chronicity of inflammation is dependent on the production of different proteases, ROS and RNS that lead to tissue damage, cell proliferation, and fibrosis during an inflammatory response [24].

*In vitro* toxicological studies use broad analyses in determining cell viability and cytotoxicity resulting from exposure to chemical substances. As a result, the establishments from *in vitro* cytotoxicity assays may be employed in predicting the possible human toxicities [16]. Steenkamp and Gouws [26] highlighted that various cell lines exhibit dissimilar sensitivities towards plant extracts.

Therefore, the current study aimed to profile the bioactive compounds in methanolic extract of *M. balsamina* leaves using molecular spectroscopic (UV-Vis and FTIR) and hyphenated chromatographic (UHPLC-qTOF-MS) techniques as well as evaluating the biological activities of the extract.

## 2. Materials and Methods

### 2.1. Materials

Analytical grade quality chemicals were used and obtained from a variety of international suppliers. Briefly, UHPLC/MS grade-quality acentronile (Romil, MicroSep, Milford, Massachusetts, USA) and methanol (Romil, MicroSep, Milford, Massachusetts, USA) were used. All solvents used for preliminary phytochemical screening were purchased from Sigma Aldrich (Saint Louis, Missouri, USA). Melphalan was purchased from (GlaxoSmithKline, Brentford, UK) and Griess reagent from (Roche diagnostics, Basel, Switzerland). Anti-inflammatory and cytotoxicity assays were performed at Prof. Van Venter's laboratory (BioAssaix, Nelson Mandela Metropolitan University, Gqeberha South Africa).

### 2.2. Extraction of Metabolites

The leaves were separated from the twigs and dried at room temperature and subsequently ground into fine powder using a mechanical grinder (Retsch Cutting Mill SM 100, Haan, Germany, Europe). The dried powdered sample was then sealed and kept in a dry area till use for further analysis [27]. Extraction was conducted according to a method described by Makita et al. [21]. Briefly, a mass of 2 g of the powdered leaf sample was weighed, and 20 ml of 80% methanol was used for extraction and was sonicated for one hour using an ultrasonic cleaning bath (SB-120DT, Loyal Key Group, Hong Kong). Then, centrifugation was done at 3000 rpm (Thermofisher, Waltham, MA, USA) for 10 minutes at room temperature (25°C) to collect the supernatant or eradicate the debris from the homogenate. The supernatant was then dried to at least 2 ml of extract using a rotary evaporator under reduced pressure at 55°C. The extract was then poured into 2 ml cryotubes and further dried overnight at constant air flow in fume hood at 40°C. Reconstitution of the dried extract was done in 1 ml of 50% MeOH, and 0.22 *μ*m nylon filters were used for filtration. The extract was stored in a freezer at −20°C to avoid degradation until they were used in other assays [21].

### 2.3. Preliminary Phytochemical Screening

Preliminary phytochemical screening tests for cardiac glycosides, flavonoids, phlabotannins, steroids, saponins, terpenoids, and tannins were conducted using methods described by Borokini and Omotayo [28] and Nemudzivhadi and Masoko [29]

### 2.4. UV-Vis Analysis

The extract was centrifuged at 3000 rpm for 10 minutes to collect the supernatant or remove the debris from the homogenate [21]. The supernatant liquid was then diluted to 1 : 10 with the same solvent. Dilutions were done in 2 ml cryotubes, and the extract was then transferred into 96-well plates. The extract was scanned in wavelength ranging from 200 to 800 nm using a SpectraMax M3 spectrophotometer (Molecular Devices, California, USA). The distinctive peaks of the UV-Vis were detected, and their values were recorded.  Table 1 shows the wavelength ranges representing specific secondary metabolites.

### 2.5. FTIR Analysis

The extract (2 g) was resuspended in 2 ml of the same solvent; this was done in 2 ml cryotubes. A vortex was used to allow the extract to solubilize, and the tube was placed on shaker for about an hour to allow further solubilisation. The extract was then analysed using ATR-IR FTIR (Alpha 1; Bruker, Germany, Europe). In order to obtain IR spectra, the extract was analysed using the standard procedure in the scanning wave number ranging from 4000 to 500 cm^−1^ with a resolution of 4 cm^−1^. Interpretation of IR spectra obtained from extract was achieved by comparing the spectral data with references from identification of functional groups existing in the leaf sample [33–35].

### 2.6. UHPLC-qTOF-MS Analysis

Ultrahigh-performance liquid chromatography and mass spectroscopy were employed for further profiling of phytoconstituents of *M. balsamina*. LC-QTOF-MS. Model LC-MS 9030 instrument utilizing Shim Pack Velox C18 column (100 mm × 2.1 mm with particle size of 2.7 *μ*m) (Shimadzu, Kyoto, Japan) was used to analyse 1 *μ*l of the extract, which was placed in a column oven set at a temperature of 40°C. A binary solvent system composed of solvent A, 0.1% formic acid in water, and solvent B, 0.1% formic acid in acetonitrile, was utilized at a flow rate of 0.4 mL/min. Analytes were chromatographically separated through a 53-minute long gradient method composed of these steps: initially, 10% B for 3 minutes; following this was a step gradient to 60% B above 37 minutes and detained at 60% B for 3 minutes; following this was another gradient to 90% B for 2 minutes, an isocratic detain at 90% for 3 min. Finally, the initial conditions (10% B) were reestablished conditions in 2 minutes, and the column was reequilibrated for a next run at 10% B for 3 minutes.

MS detection parameters were set in the following manner: negative electrospray ionization (ESI) modes; an interface voltage of 3.5 kV; nebulizer gas flow at 3 L/min; heating gas flow at 10 L/min; temperature of heat block at 400°C; CDL temperature at 250°C; voltage of detector at 1.70 kV and temperature of TOF tube at 42°C. Acquisition of high accurate mass with mass error below 1 ppm was ensured by using sodium iodide (NaI) as a mass calibration. For both high-resolution MS and tandem MS (MS/MS) experiments, an *m/z* ranging from 100 to 1000 was employed. For MS/MS experiments, argon gas was utilized as a collision gas, and to generate possible fragments, MS^E^ mode utilizing a collision energy ramp of 15 to 25 eV was required.

### 2.7. *In Vitro* Cytotoxicity Screening of the *M. balsamina* Methanolic Extract

A mass of 0.02 g of extract was weighed, and dimethyl sulfoxide (DMSO) was used as a reconstitution solvent to give a final concentration of 100 mg/mL, followed by sonication of the sample to completely dissolve the extract, and then, it was stored at 4°C for further use. The human colorectal adenocarcinoma cell lines (HT29 and Caco2) and African green monkey kidney cells (Vero cells) were used for cytotoxicity screening. These were maintained in 10 cm culture dishes inside a humidified incubator (Thermofisher, Waltham, MA, USA) with 5% CO_2_ at 37°C. The constituents used for the complete growth medium were Dulbecco's modified Eagle medium (DMEM) supplemented with 10% foetal bovine serum (FBS) and 10% penicillin-streptomycin for the three cell lines.

Cells were seeded into 96-well microtiter plates at a density of 4000 cells/well using a volume of 100 *μ*l in each well. For cell attachment, the cells were left overnight at 37°C, 5% CO_2_, and 100% relative humidity. Cells were treated with 50, 100, and 200 *μ*g/ml of extract. Melphalan, a toxic agent as highlighted by Sigidi et al. [36], was used as a positive control, and volumes of 10, 20, and 40 *μ*M were diluted in culture medium. Cells were then further treated with 100 *μ*L aliquots of the diluted extract in the fresh medium and incubated again for 48 hours. The treatment medium was aspirated from all wells, and 100 *μ*L of Hoechst 33342 nuclear dye (5 *μ*g/mL) was added to each well, followed by incubation for 20 minutes at room temperature (25°C).

Propidium iodide (PI) was used at 100 *μ*g/mL to stain the cells, and this was done for enumeration of the proportion of dead cells within the population. An image of the cell was then captured immediately after PI was added using the ImageXpress Micro XLS Widefield Microscope (Molecular Devices San Jose, California, USA) with a 10x Plan Fluor objective and DAPI and Texas Red filters cubes. For each well, nine images were acquired as a representative of 75% of the surface area of the well. For quantifying viable and dead cells, a screening assay was performed, and acquired images were analysed using the MetaXpress software and Multiwavelength Cell Scoring Application Module.

### 2.8. Anti-Inflammatory Activity of the *M. balsamina* Leaf Extract

*Momordica balsamina* extract was dissolved in DMSO to give a final concentration of 100 mg/ml and diluted further into culture medium. A total of 100 *μ*M of aminoguanidine was used as a positive control since it is known as an inhibitor of nitric oxide as highlighted by Sigidi et al. [36]; hence, it was employed as an indicator for anti-inflammatory activity.

RAW 264.7 cells were seeded at a density of 1 × 10^5^ cells/well into 96-well plates and cell attachment took place overnight in a Heracell VIOS CO_2_ Incubator (Thermofisher, Waltham, MA, USA). Samples were then diluted in DMEM after removal of spent culture medium. These samples were added in volumes of 50 *μ*l in each well to give final concentrations of 25, 50, 100, and 200 *μ*g/mL. The corresponding wells were filled with 50 *μ*l of LPS containing medium to give a final concentration of 500 *μ*g/mL, and this was done for assessment of anti-inflammatory activity.

#### 2.8.1. Nitrite Production

Aminoguanidine was utilized as a positive control, and cells were further incubated for 18 hours. To quantify NO production, a new 96-well plate was used, and 50 *μ*l of spent culture medium was transferred and 50 *μ*l Griess reagent was also added. Measurement of absorbance (VersaMax ELISA Microplate Reader, Sunnyvale, CA; USA) was done at 540 nm, and the results were expressed relative to the appropriate untreated control. For the determination of NO concentration in each sample, a standard curve using sodium nitrite dissolved in culture medium was used.

#### 2.8.2. Assessment of Cell Viability

MTT was used to assess cell viability and confirm the absence of toxicity as a contributory factor. This was done by removing the remaining medium and treating each well using a medium comprising of 0.5 mg/ml MTT as a replacement and incubating for 30 minutes at 37°C. MTT was then eradicated and 200 *μ*L of DMSO was added to each well to dissolve the formazan crystals. Absorbance was measured at 540 nm using a spectrophotometer (BioTek® PowerWave XS, Winooski, VT, USA).

## 3. Results and Discussion

A range of techniques have been employed to profile bioactive compounds in plants, and many reports have shown that plants contain numerous secondary metabolites. In this study, molecular spectroscopic (FTIR and UV-Vis) and hyphenated chromatographic (UHPLC-qTOF-MS) techniques were used for plant profiling.

### 3.1. Preliminary Phytochemical Screening

Phytochemical screening tests revealed the presence of tannins, phlobatannins, cardiac glycosides, terpenoids, saponins, and flavonoids with an exception of steroids in the methanolic extract as shown in Table 2.

The findings in this study correspond to the results obtained in a study by Adamu et al. [11], who also detected the presence of tannins, flavonoids, saponins, and glycosides in methanolic extract of *M. balsamina* leaves. However, the difference with the current study is due to the absence of terpenoids. This dissimilarity could be an outcome of geographical and ecological differences [11, 37]. The outcomes were also different to other studies that detected steroids in the fruit pulp of *M. balsamina* [1,5].

According to Shrestha et al. [38], the presence of cardiac glycosides could mean that the plant is capable of lowering blood pressure. Terpenoids have been reported to exhibit several biological activities, such as anti-inflammatory [23], immunomodulatory, and antimicrobial activities [38, 39]. Flavonoids and tannins have been reported as free radical scavenging molecules [16, 40, 41]. Saponins are known to possess a significant ability to precipitate and coagulate red blood cells [39] and exhibit anti-inflammatory activity [16].

### 3.2. UV-Vis Analysis

**Figure 1:**
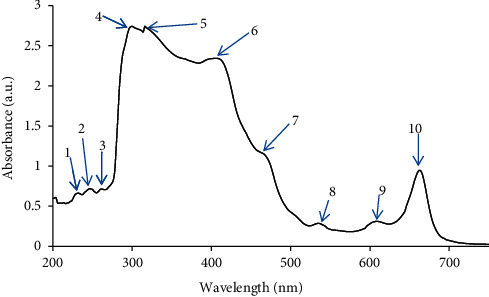
UV-visible spectral analysis of the methanolic extract of *M. balsamina* selected from 200 nm to 700 nm due to broadness of distinctive peaks and proper baseline with each arrow indicating characteristic peaks.

The UV-Vis profile of the methanolic extract (Figure 1; Table 3) of *M. balsamina* was selected from 200 nm to 750 nm due to broadness of distinctive peaks and proper baseline. This technique was used to detect the presence of phytochemicals by identifying compounds containing *π*-bonds, lone pairs of electrons, *σ-*bonds, aromatic rings, and chromophores in the UV-Vis region on the electromagnetic spectrum ranging from 200 to 750 nm.

The UV-Vis profile (Figure 1) revealed the presence of 10 peaks at 226, 240, 256, 296, 312, 404, 462, 530, 604, and 658 nm with absorption ranging from 0.3 to 2.7 a.u., as shown in Table 3. In previous studies, absorption bands that occur at 234–676 nm are characteristic for alkaloids, flavonoids, and phenolic compounds [17, 30]; in this case, all peaks occur in this range. This therefore suggests the presence of these secondary metabolites in the extract.

The peaks 226, 240, 256, and 312 were identified as flavonoids and their derivatives and this was due to their occurrence ranging from 230 to 285 (band I) nm and from 300 to 350 (band II) nm [18, 31, 32]. The presence of aromatic rings and other rings is the reason behind the two absorption spectra for flavonoids and phenolic compounds [17]. In another study, it was also highlighted that the occurrence of peaks ranging from 280 to 330 nm is characteristic for the phenolic derivatives [19], thus indicating the presence of phenolic compounds in the extract in the current study.

The peak 404 was also detected in this study; a previous study highlighted that peaks occurring at 400–450 nm indicate the presence of carotenoids [17]. The peaks 404 and 462 were also identified, and these were characteristic for tannins due their occurrence at 350–500 nm [17]. The peaks 404, 462, and 530 were characteristic for terpenoids, and this is due to occurring at 400–550 nm [18, 32]. The peaks 604 and 658 were identified as chlorophyll, and this is due to their occurrence at 600–700 nm [18, 19, 32].

### 3.3. FTIR Analysis

**Figure 2:**
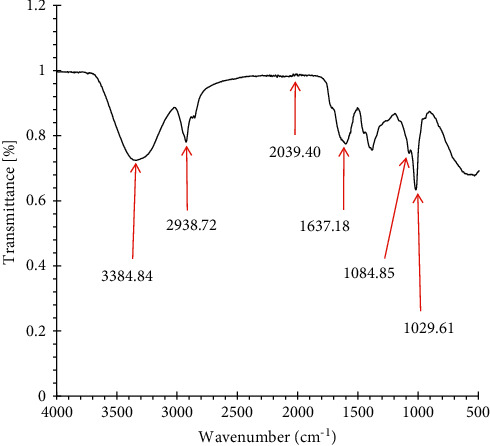
FTIR spectrum analysis of the methanolic extract of *M. balsamina* with each arrow showing distinctive peaks' characteristic for various functional groups indicating specific phytochemical compounds.

FTIR spectral analysis was used to detect functional groups of compounds present in *M. balsamina*, and this was based on peak values in the region of infrared radiation. When the extract was passed into FTIR, the functional groups of the compounds were separated based on their peak ratios [18]. The FTIR spectral analysis of the methanolic extract (Figure 2) as well as the peak values and functional groups (Table 4) are displayed as follows.

The FTIR spectral analysis of methanolic extract detected the presence of functional groups in six different frequency ranges (Table 4). The FTIR spectrum (Figure 2) showed six major peaks at 3384.84 cm^−1^, 2938.72 cm^−1^, 2039.40 cm^−1^, 1637.18 cm^−1^, 1084.85 cm^−1^, and 1029.61 cm^−1^.

Since aqueous methanol was used to extract the sample, there could be a methanol band in the spectrum (Figure 2). Thus, characteristic methanol band would be 3384.84 cm^−1^ showing stretching vibration of O-H group or O-H wagging of phenolic compounds [42]. This therefore strongly suggests the presence of phenolic compounds, which have been reported to exhibit antioxidant activities [41].

The analysis revealed the presence of 10 various functional groups belonging to six different compounds as shown in Table 4.

The band occurring at 3384.84 cm^−1^ indicates that alkaloids may be present which could be due to the N–H stretch [31, 43]. The identified band at 2039.40 cm^−1^ could be due to the presence of alkynes, and this is attributed to the C≡C stretching vibrations. The stretching vibration of C–H band at 2938.72 cm^−1^ could be ascribed to the presence of CH_2_ and CH_3_ group, which indicates the presence of terpenes [31, 43].

The band at 1637.18 cm^−1^ could be attributed to the presence of a deformed aromatic ring, amino acids, flavonoids, and stretching vibrations of C=C groups [33]. The identified bands at 1084.85 cm^−1^ and 1029.61 cm^−1^ could be due to presence of C–O stretching vibration due to an ester group or secondary alcohol [19].

Fourier transform infrared transmission is very useful in plant characterization because it reveals the presence of inorganic and organic compounds in plants. The presence of functional groups serves as an indicator of different medicinal properties or biological activities of *M. balsamina* leaves.

### 3.4. UHPLC-q-MS Analysis

**Figure 3:**
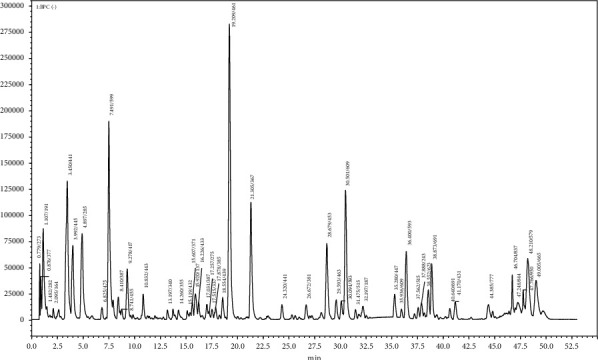
Representative UHPLC-qTOF-MS chromatogram showing metabolites present in the methanolic extract of *M. balsamina.*

**Figure 4:**
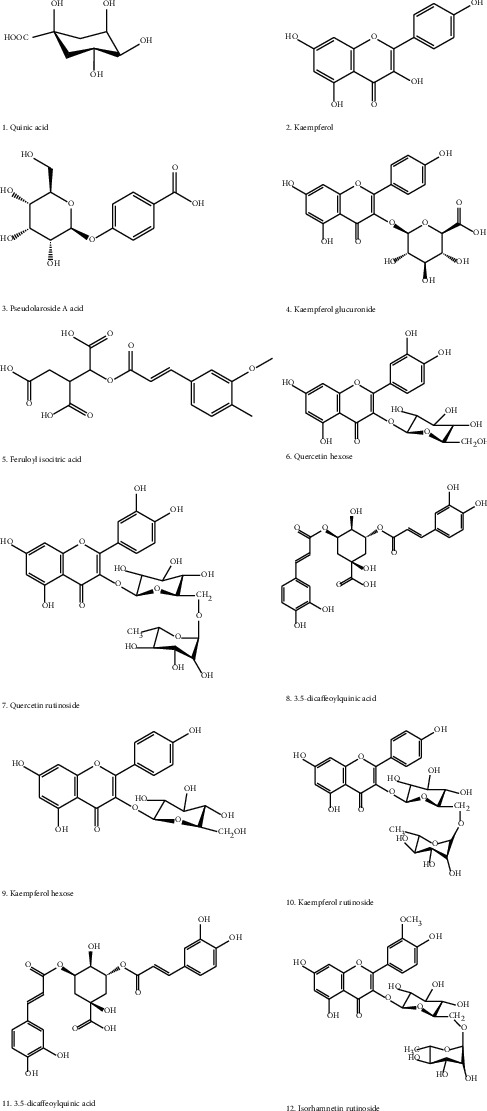
Chemical structures (1–12) of metabolites isolated from the methanolic extract of *M. balsamina* indicating the presence of flavonoids and acids.

Metabolite extraction and profiling of *M. balsamina* methanolic extract was carried out subsequently. Analyses were done using an LC-qTOF-MS operating in negative electrospray ionization (ESI) mode. From the UHPLC-qTOF-MS chromatogram (Figure 3), a total of 12 chromatographic peaks showing various metabolites (Figure 4) were identified as shown in Table 5.

The most common flavonoids are kaempferol, quercetin, and isorhamnetin, and these abundantly exist as glycosides in the plant's tissues [21]. Flavonoids with these aglycones have been reported to exhibit a wide range of health-promoting activities, such as inhibition of inflammation [44, 45]. Hence, two quercetin-O-glycosides, four kaempferol-O-glycosides, and one isorhamnetin-O-glycoside were identified in this study.

Quercetin has been reported as a crucial dietary flavonoid that is associated with plethora properties capable of suppressing certain ailments linked to chronic diseases [21]. According to Gbashi et al. [46], this flavonoid aglycone has been suggested as a hepatoprotective agent. Quercetin rutinoside (molecule **7**) with a precursor ion at *m/z* 609 [M-H]^−^ was identified in this study. Other studies highlighted that quercetin rutinoside, known as rutin, has high antioxidant activity potential attributing to biological activities such as protection of liver cells and suppression of haemoglobin oxidation [21, 22]. Furthermore, the molecule has also been reported as an anti-inflammatory agent making it useful in the treatment of chronic diseases [21]. Quercetin hexose (molecule **6**) with a precursor ion at *m/z* 463 [M-H]^−^ was also detected in current study [21].

Research has shown that kaempferol is of great significance in managing cancer-associated ailments [21, 47] as well as inhibiting oxidative stress [46]. Molecule **10** was identified as kaempferol rutinoside, with a precursor ion at *m/z* 593.1496 [M-H]^−^ [2]. Molecule **9** was identified as kaempferol hexose [2, 44, 46], with a precursor ion at *m/z* 447 [M-H]^−^. Molecule **2** was identified as kaempferol, with a precursor ion at *m/z* 285 [M-H]^−^. Molecule **4** was identified as kaempferol glucuronide [46], with a precursor ion at *m/z* 461.

Isorhamnetin is a methylated form of quercetin [21], and has been reported to exhibit an anti-inflammatory activity [48]. This flavonoid aglycone has been proven as an antioxidant agent [44, 49]. Molecule **12** was identified as isorhamnetin rutinoside, with a precursor ion at *m/z* 623 [M-H]^−^ [21].

Chlorogenic acids (CGA) are secondary metabolites found in plants and are of great significance because they possess a variety of health benefits, which include anti-inflammatory and antidiabetic activities [3, 50]. Madala et al. [3] defined CGA as a molecule that is formed from an ester bond between single or multiple cinnamic acids (*p-*coumaric, caffeic, and ferulic acid) and quinic acid, resulting in *p-*coumaroylquinic acid, caffeoylquinic acid, and feruloylquinic acid. A total of three chlorogenic acids were identified, namely, quinic acid with a precursor ion at *m/z* 191 [M-H]^−^ and two isomers of dicaffeoylquinic acids (molecules **8** and **11**), with a precursor ion at *m/z* 515 [M-H]^−^ eluted at different retention times, 31.475 and 37.562, respectively (Figure 3; Table 5).

Hydroxyl-cinnamic acids (HCAs) have been reported to exist as positional and geometric isomers conjugated to various organic acids, namely, quinic and isocitric acids [51, 52]. The formation of hydroxycinnamoyl isocitric acid is due to the formation of a conjugate between HCA derivatives and organic acids, and this includes the esterification between one of the derivatives of HCA and an isocitric acid. This can occur at position 2 (C2) [51]. Masike et al. [51] further highlighted that derivatives of hydroxycinnamoyl isocitric acid are not well documented, and this is ascribed to misidentification of these compounds with monoacyl chlorogenic acids because they have the same molecular mass of conjugates, namely, caffeoyl- (354 Da), p-coumaroyl- (338 Da), and feruloyl- (368 Da). In this study, feruloyl isocitric acid [51] was identified at precursor ion *m/z* 367 [M-H]^−^ (molecule **5**) as predicted by the accurate high-resolution mass spectrometer (LC-QTOF-MS model LC-MS 9030 instrument). To the best of our knowledge, this is the first report on the presence of feruloyl isocitric acid in *M. balsamina* leaves.

A peculiar compound that has not yet been documented in relation to compounds isolated in *M. balsamina* was identified in the current study. Pseudolaroside A acid (molecule **3**: C_13_H_16_O_8_) of precursor ion *m/z* 299 [M-H] ^–^ was identified as a dimer of (C_26_H_32_O_16_) at precursor ion *m/z* 599 [M-H]^−^. This benzoic acid allopyranoside was isolated from the bark of *Pseudolarix kaempferi* as a colourless amorphous solid by Lui et al. [53]. Kim et al. [54] also isolated this compound from the roots of *Coix lachrymal-jobi var. mayuen.* To the best of our knowledge, only a few studies have been done on this compound, and its significance is yet to be documented.

Nagarani et al. [4] reported other flavonoid molecules in other *Momordica* species, such as catechin, chlorogenic acid, caffeic acid, and ferulic acid. Previous studies highlighted that these species, namely, *M. balsamina, M. charantia*, and *M. foetida*, are abundant sources of flavonoids of different forms, and these include a variety of isomers of the quercetin-, kaempferol-, and isorhamnetin-*O-*glycosides [2, 44, 55]. In another study by Madala et al. [3], the results revealed that *Momordica* species are comprised of all forms of common cinnamic acids, such as caffeic, *p*-coumaric, and ferulic acid. In addition, 4-acylated quinic acids were identified: 4-*pCo*QA, 4-CQA, and 4-FQA.

#### 3.4.1. *In Vitro* Cytotoxicity Screening of the Methanolic Extract of *M. balsamina* against Human Colorectal Adenocarcinoma (Caco2 and HT29), Vero, and RAW 264.7 Cell Lines

**Figure 5:**
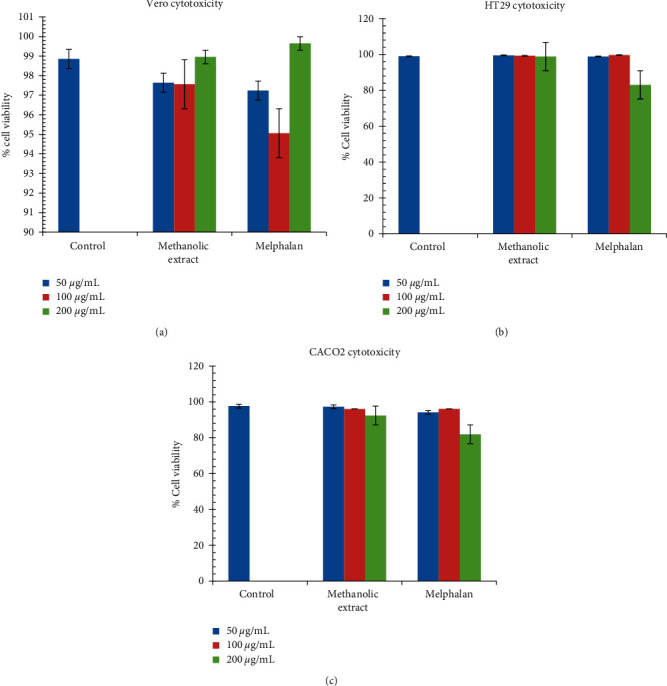
Cytotoxicity analysis of the methanolic extract of *M. balsamina* against three cell lines, namely, Vero cytotoxicity (a), HT29 cytotoxicity (b), and Caco2 cytotoxicity (c), at 3 concentrations. Melphalan was employed as a positive control.

The cytotoxic effects of the extract against Caco2, HT29, and Vero cell lines were assessed and evaluated using MTT assay. In all the three cell lines tested, no cytotoxic effect was observed. The extract exhibited no cytotoxicity in Caco2, HT29, RAW 264.7, and Vero cell lines (Figures 5(a)–5(c), resp.).

Medicinal plants may be considered effective in clinical applications if the preparations show selective toxicity to the targeted microorganism [56]. The findings confirmed that *M. balsamina* leaves have no cytotoxic activity against human colorectal adenocarcinoma cell lines HT29 and Caco2. The methanolic extract showed less toxicity compared with melphalan standard as presented in Figure 5(a). Cell viability at the highest concentration (200 *μ*g/ml) of the extract was above 98%, which is comparable with cells, only control. Based on the visual inspection of cell viability of extract against all cell lines (Figures 5(a)–5(c)), cell viability was not affected or did not decrease even in the presence of the extract, strongly suggesting that these leaves are safe for consumption.

According to a study done by Ramalhete et al. [57], *M. balsamina* compounds showed no cytotoxicity against human breast cancer (MCF-7) cell lines. Furthermore, *in vivo* studies have demonstrated that *M. balsamina* extracts have shown an extremely weak or inactive toxicity [7, 57]. In another study by Ramalhete et al. [58] on the activity of *M. balsamina* against bacterial efflux pumps, the extracts showed no toxicity against human lymphocytes [58]. These findings support the observed lack of toxicity of *M. balsamina* extracts despite the diversity of the phytochemicals detected.

#### 3.4.2. *In Vitro* Anti-Inflammatory Screening of the Methanolic Extract of *M. balsamina* on RAW 264.7 Cell Lines

**Figure 6:**
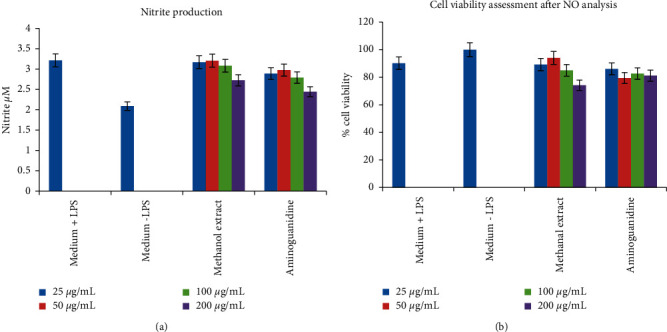
Anti-inflammatory analysis of the *M. balsamina* methanolic extract in RAW 264.7 cell lines. (a) The effect of the methanolic plant extract on the production of nitrate and cell viability in LPS-stimulated and unstimulated RAW macrophages. (b) Aminoguanidine, an inhibitor of iNOS expression serving as a positive control to confirm the functionality of the assay.

The *in vitro* anti-inflammatory potential of *M. balsamina* methanolic extract was evaluated on RAW 264.7 cells. The observed results (Figures 6(a) and 6(b)) indicated that the methanolic extract at the highest concentration (200 *μ*g/ml) exhibited potential anti-inflammatory activity. This is due to the presence of anti-inflammatory exhibiting compounds, such as flavonoids and phenolic compounds, as identified by the phytochemical analyses conducted in this study. In support of the results obtained in this study, Nagarani et al. [4] highlighted that *Momordica* species have potential anti-inflammatory activity, and it is possible that the anti-inflammatory effects may be correlated to the phytochemical composition of the plant.

A study by Thakur et al. [5] revealed that the methanolic extract of *M. balsamina* exhibited anti-inflammatory activity and this is in agreement with the current study. Sigidi et al. [36] defined NO as an intracellular free radical that can be generated in different mammalian cells. This molecule plays a role in neurotransmission, acute and chronic inflammation, and host defense mechanisms against different pathogenic microbes. However, it may induce a toxic reaction against the host's tissues if produced in higher levels [36, 56].

In this study, flavonoids were detected in the extract and have been reported as naturally occurring anti-inflammatory agents [59]. In support of that statement, a few studies have reported flavonoids to have anti-inflammatory properties through inhibition of transcription factors and regulatory enzymes that play a crucial role in controlling inflammatory mediators [24, 25]. Therefore, flavonoids exhibit an anti-inflammatory activity. It is important to highlight that anti-inflammatory activity of *M. balsamina* extract may be linked to the presence of flavonoids.

Qualitative and quantitative analyses of the phytochemical composition and toxicological effects of *Momordica* leaves are pivotal because these leaves can be suitable for incorporation into functional food in consideration of traditional and scientific knowledge of diverse assessments [4].

## 4. Conclusions

In this study, it was confirmed that *Momordica balsamina* contains a wide variety of secondary metabolites that could be of medicinal importance. The study also demonstrated that the leaves could be used as anti-inflammatory agents and are nontoxic to the colorectal adenocarcinoma cell lines, thus making them safe for consumption. This is the first study to report pseudolaroside A acid and feruloyl isocitric acid in *Momordica balsamina* leaves. Further investigations on these compounds and their significances are necessary.

## Figures and Tables

**Table 1:** Wavelength ranges representing specific secondary metabolites.

Absorption maxima (wavelength ranges)	Phytochemical compounds (metabolites)	References
234–676 nm	Flavonoids, alkaloids, phenolic compounds	[17, 30]
230–285 nm (band I)	Flavonoids and their derivatives	[31]
230–290 nm (band I)	Flavonoids	[18, 19, 32]
300–350 nm (band II)	Flavonoids and their derivatives	[18, 31, 32]
350–500 nm	Tannins	[17]
400–450 nm	Carotenoids	[17]
400–550 nm	Terpenoids	[18, 19, 32]
600–700 nm	Chlorophyll	[18, 19, 32]

**Table 2:** Preliminary phytochemical screening profile of the *M. balsamina* extract.

Metabolites	Methanolic extract
Cardiac glycosides	+
Flavonoids	+
Phlobatannins	+
Steroids	−
Saponins	+
Tannins	+
Terpenoids	+

(+): present; (−): absent

**Table 3:** UV-Vis spectrum peak values of the methanolic extract of *M. balsamina*.

S. no.	Wavelength (nm)	Absorbance (a.u.)
1	226	0.6
2	240	0.7
3	256	0.7
4	296	2.7
5	312	2.7
6	404	2.3
7	462	1.2
8	530	0.3
9	604	0.3
10	658	0.9

**Table 4:** FTIR peak values and functional groups in methanolic extracts of *M. balsamina* [31, 33, 42, 43].

No.	Frequency ranges (cm^−1^)	Frequency peak values (cm^−1^)	Vibration/bond	Specific functional group	Chemical compound
1	3600–3200	3384.84	O–H stretch	Alcohols, phenols (hydrogen bonding)	Aromatic
	3400–3250	3384.84	N–H stretch	1°, 2° amines and amides	Amines and amides
2	3000–2850	2938.72	C–H stretch	Alkanes	Aliphatic
3	2270–1940	2039.40	C≡C stretch	Alkynes	Aliphatic
4	1680–1620	1637.18	C=C stretch	Alkene	Aliphatic
5	1320–1000	1084.85	C–O stretch	Alcohols, carboxylic acids, esters, and ethers	Acid and alcohol
6	1320–1000	1029.61	C–O	Alcohols, carboxylic acids, esters, and ethers	Acid and alcohol

**Table 5:** UHPLC-qTOF-MS profile of metabolites isolated from the leaf extracts of *M. balsamina*.

	Metabolite	Elemental composition	Rt (min)	[M-H]
1	Quinic acid	C_7_H_12_O_6_	1.107	191
2	Kaempferol	C_15_H_10_O_6_	4.897	285
3	Pseudolaroside A (dimer)	C_26_H_32_O_16_	7.491	599
		Dimer: C_13_H_16_O_8_		Dimer: 299
4	Kaempferol glucuronide	C_21_H_18_O_12_	19.209	461
5	Feruloyl isocitric acid	C_16_H_16_O_10_	21.305	367
6	Quercetin hexose	C_21_H_20_O_12_	29.5923	463
7	Quercetin rutinoside	C_27_H_30_O_16_	30.501	609
8	Dicaffeoylquinic acid isomer I	C_25_H_24_O_12_	31.475	515
9	Kaempferol hexose	C_21_H_20_O_11_	35.936	447
10	Kaempferol rutinoside	C_27_H_30_O_15_	36.400	593
11	Dicaffeoylquinic acid isomer II	C_25_H_24_O_12_	37.562	515
12	Isorhamnetin rutinoside	C_27_H_30_O_16_	38.552	623

## Data Availability

The supporting data can be provided from the corresponding author upon request.
